# Malignancy rates and diagnostic performance of the Bosniak classification for the diagnosis of cystic renal lesions in computed tomography – a systematic review and meta-analysis

**DOI:** 10.1007/s00330-016-4631-9

**Published:** 2016-10-19

**Authors:** Sabina Sevcenco, Claudio Spick, Thomas H. Helbich, Gertraud Heinz, Shahrokh F. Shariat, Hans C. Klingler, Michael Rauchenwald, Pascal A. Baltzer

**Affiliations:** 1 Department of Urology, Medical University of Vienna, Währinger Gürtel 18-20, 1090 Vienna, Austria; 2 Department of Biomedical Imaging and Image-guided Therapy, General Hospital Vienna, Medical University of Vienna, Währinger Gürtel 18-20, A-1090 Vienna, Austria; 3 Department of Radiology, University Hospital of Sankt-Pölten, Propst-Führer-Straße 4, 3100 St., Pölten, Austria; 4 Department of Urology, Wilhelminenspital, Montleartstraße 37, 1160 Vienna, Austria; 5 Department of Urology, Donauspital, Langobardenstraße 122, 1220 Vienna, Austria

**Keywords:** Bosniak classification, Renal cysts, Kidney cancer, Meta-analysis, Systematic review

## Abstract

**Objective:**

To systematically review the literature on the Bosniak classification system in CT to determine its diagnostic performance to diagnose malignant cystic lesions and the prevalence of malignancy in Bosniak categories.

**Methods:**

A predefined database search was performed from 1 January 1986 to 18 January 2016. Two independent reviewers extracted data on malignancy rates in Bosniak categories and several covariates using predefined criteria. Study quality was assessed using QUADAS-2. Meta-analysis included data pooling, subgroup analyses, meta-regression and investigation of publication bias.

**Results:**

A total of 35 studies, which included 2,578 lesions, were investigated. Data on observer experience, inter-observer variation and technical CT standards were insufficiently reported. The pooled rate of malignancy increased from Bosniak I (3.2 %, 95 % CI 0–6.8, I^2^ = 5 %) to Bosniak II (6 %, 95 % CI 2.7–9.3, I^2^ = 32 %), IIF (6.7 %, 95 % CI 5–8.4, I^2^ = 0 %), III (55.1 %, 95 % CI 45.7–64.5, I^2^ = 89 %) and IV (91 %, 95 % CI 87.7–94.2, I^2^ = 36). Several study design-related influences on malignancy rates and subsequent diagnostic performance indices were identified.

**Conclusion:**

The Bosniak classification is an accurate tool with which to stratify the risk of malignancy in renal cystic lesions.

***Key points*:**

• *The Bosniak classification can accurately rule out malignancy*.

• *Specificity remains moderate at 74* % (*95* % *CI 64*–*82*).

• *Follow*-*up examinations should be considered in Bosniak IIF and Bosniak II cysts*.

• *Data on the influence of reader experience and inter*-*reader variability are insufficient*.

• *Technical CT standards and publication year did not influence diagnostic performance*.

**Electronic supplementary material:**

The online version of this article (doi:10.1007/s00330-016-4631-9) contains supplementary material, which is available to authorized users.

## Introduction

Contrast-enhanced computed tomography (CT) is considered the imaging standard for the evaluation of renal cysts. Since its introduction, the Bosniak classification for cystic renal masses has found widespread acceptance. This is due to its simple structure, with a low number of diagnostic categories, each of them associated with a suggestion for clinical management [1, 2] (Table 1). Bosniak category I and category II lesions are simple and minimally complex cysts and require no further work-up. A Bosniak category III lesion is an indeterminate complex cyst with an increased probability of malignancy ranging from 31 % to 100 %. For these cysts, the usual workup is surgery or, in selected cases, radiological follow-up [2]. Bosniak IV cysts have clearly malignant features and surgical therapy is recommended. In order to decrease unnecessary surgical interventions, a fifth category, Bosniak IIF has been introduced. This category is a modification of the initial Bosniak classification and describes a group of minimally complex cystic lesions, separate from Bosniak II and III, for which short-term (3–6 months) imaging surveillance is recommended [3] (Table 1).

**Table 1:** The Bosniak classification for evaluation of renal cysts [2]

Bosniak category	Imaging features	Work-up
I	Simple benign cyst: hairline-thin wall without septa, calcifications, or solid components. Density similar to water (≤15 HU), no enhancement after IV contrast medium administration.	Benign, no further work-up necessary
II	Benign cyst with minimal complicated features: may present with a few hairline-thin septa, fine calcifications in wall or septa. Further homogeneous high-attenuation (>15 HU) lesions <3 cm in size, sharp margins without enhancement	Benign, no further work-up necessary
IIF	Similar to II but more complicated features: more hairline-thin septa, minimal enhancement of septum or wall. Further minimal thickening of septa/wall. Calcifications may be nodular/thick. No enhancing renal mass. Also, non-enhancing, high attenuation (>15 HU) lesions >3 cm. Circumscribed margins	Probably benign, Follow-up recommended
III	Complicated cystic masses with thickened/irregular walls or enhancing septa	Probably malignant, surgery or active surveillance recommended
IV	Enhancing renal masses with cystic/necrotic components	Malignant, surgery recommended

While in cancer diagnosis a maximum sensitivity is always desirable, false-positive findings may cause serious problems and side effects, especially in vulnerable organs like the kidneys. Evidence-based clinical decision-making requires an assessment of the accumulated empirical evidence. We noted a discrepancy between the broad application of the Bosniak classification in clinical practice and the lack of a systematic review and quantitative data synthesis demonstrating strengths and weaknesses of this clinical decision rule. While the Bosniak classification is clinically established, how accurate a positive or negative result is and whether it may be reproduced using different equipment or readers remains unknown. Through a systematic review and meta-analysis, we aimed to address the rate of malignancy in different Bosniak categories, the Bosniak classification’s diagnostic accuracy and factors that influence malignancy rates and diagnostic performance.

## Materials and methods

### Search strategy

Two readers independently performed a systematic search of the Pubmed and Scopus databases including articles listed from 1 January 1986 to 18 January 2016. The predefined search term ‘Bosniak’ was used. The title and abstracts from search results were screened and the full text of eligible studies was retrieved. Only original, peer-reviewed research articles that investigated the rate of renal cyst malignancy in adult human subjects imaged by CT and classified according to the Bosniak classification were eligible for this study. Additional backward snowballing was performed scanning the references of retrieved articles for additional studies [4].

### Study selection

**Fig. 1:**
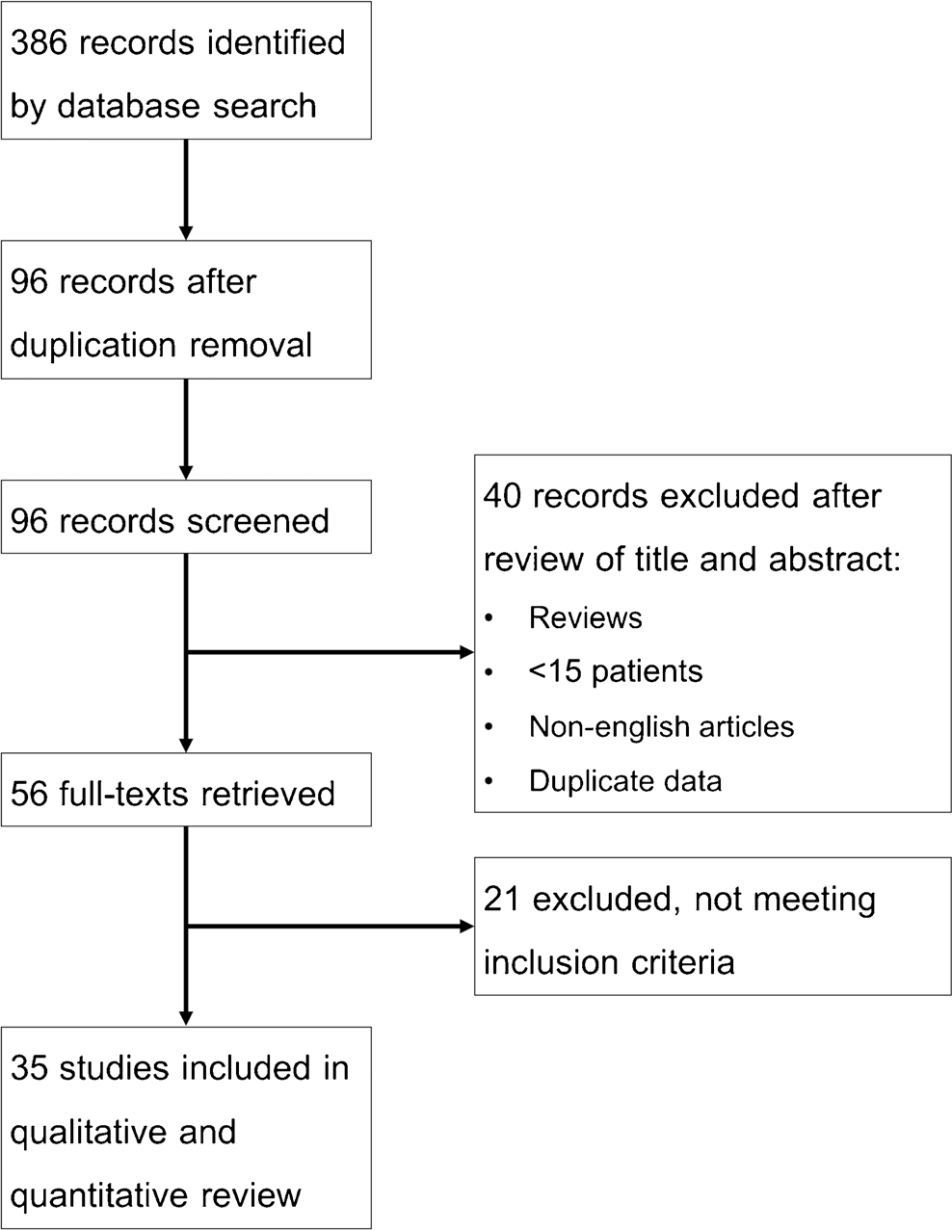
Flowchart showing the study selection process

Both reviewers independently screened all identified records for eligibility. A third arbitrator resolved any disagreement. If the title and abstract did not provide sufficient information, the full text was retrieved. Included were articles fulfilling the following conditions: (1) a reference standard had to be established either by histopathology workup or imaging follow-up; (2) eligible studies had to be published in English and (3) eligible studies had to include at least 15 patients. Study quality was assessed using the QUADAS 2 tool. The selection process by which the included studies were derived for data extraction is shown in Fig. 1.

### Data extraction

Two readers (one board-certified radiologist and one board-certified urologist with >5 years experience in renal imaging) performed raw data extraction. Afterwards, data were checked for discrepancies which were solved in consensus. A third reader controlled all data extracted by the initial readers and corrected any discrepancies in consensus with them. The following parameters were collected and entered into a spreadsheet: author name; publication year; study design (retrospective vs. prospective); number of patients and lesions; reference standard (histopathology obtained by surgery or image-guided biopsy or follow-up, duration of follow-up); reader number; experience and inter-reader agreement; and the technical parameters of CT. Numbers and final diagnosis (benign or malignant) were extracted for all Bosniak categories (I, II, IIF, III and IV). Studies were further classified as either reporting the prevalence of malignancy in certain Bosniak subgroups only or diagnostic (including both lesions of any Bosniak I, II or IIF, and Bosniak III or IV). Both readers applied QUADAS 2 items to assess study quality and likelihood of bias [5]. Again, if present, disagreement was solved in consensus. In case of disagreement, a third reader acted as an arbitrator.

### Data synthesis and analysis

Analyses were performed using the software programs Open Meta-Analyst for Mac OS Yosemite 10.10 (http://www.cebm.brown.edu/open_meta) and StataSE 12 (StataCorp, College Station, TX, USA). Raw extracted data from eligible articles were used to construct forest plots of the rate of malignancy in Bosniak categories I–IV; in addition, meta-regression was performed in order to identify a possible influence on the prevalence of malignancy by the factors listed above. In case of positive findings, the forest plot was grouped according to the respective variable identified by meta-regression. For the assessment of heterogeneity, I^2^-statistics were calculated and interpreted in accordance with the proposal of Higgins and Thompson as showing low (I^2^ around 25 %), medium (I^2^ around 50 %) or high (I^2^ around 75 %) heterogeneity [6].

The diagnostic accuracy of the Bosniak classification for the differentiation between benign and malignant renal cysts was calculated by tabulating results into positive (Bosniak III and IV) versus negative (Bosniak I, II and IIF) diagnostic test results. The reference standard for malignant and benign diagnoses was defined as the final diagnosis confirmed by histopathology and/or follow-up. For calculation of sensitivity and specificity, a diagnostic random-effects model, using the method of DerSimonian and Laird, was used. For calculation purposes, a correction factor of 0.5 was added to zero findings. A summary receiver operating characteristic (ROC) curve was constructed by using a bivariate (maximum likelihood) model. Again, data heterogeneity was assessed by I^2^-statistics.

Meta-regression was applied to investigate the possible influence of variables (sample size of the respective study, reference standard – histopathology only or histopathology and/or follow-up, study published before or after the introduction of the Bosniak IIF category, benign lesions only, including Bosniak IIF or also Bosniak I and/or II) and technical factors including slice thickness (grouped as ≤5, ≤10 or not given), detector rows (grouped as: up to 4, 16, 64 or higher and not given) or whether there was any technical information given or not on sensitivity and specificity. P-values of <0.05 were interpreted as indicating a significant result.

Finally, publication bias was assessed by construction of funnel plots and Deek’s test for funnel plot asymmetry.

## Results

### Study characteristics, bias

**Fig. 2:**
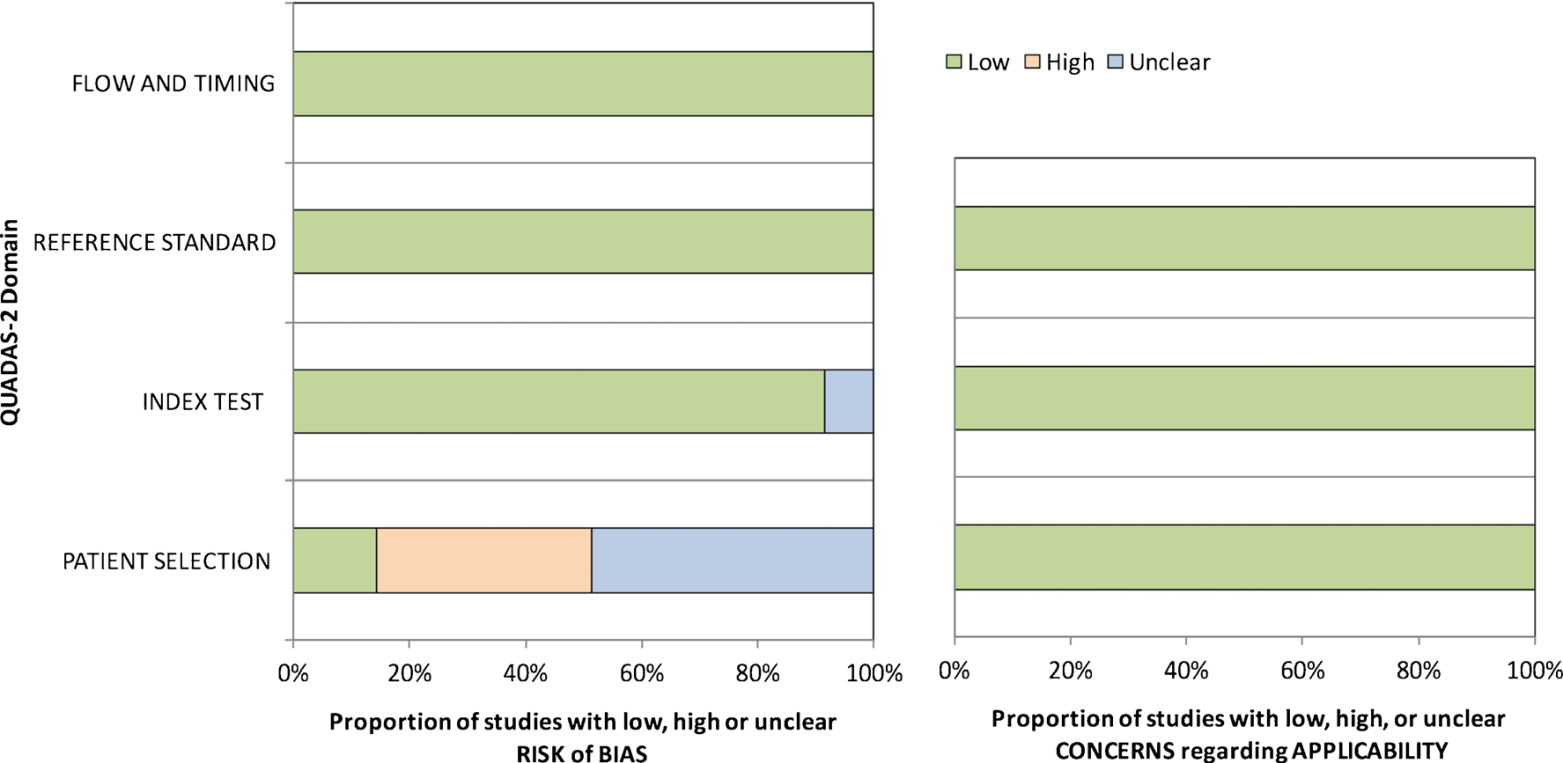
QUADAS 2 assessment results

Overall, 35 eligible studies were selected (Fig. 1, Tables 2 and 3). In our meta-analysis, a total of 2,557 patients with 2,578 lesions (862 malignant lesions, 33.4 %) were included. QUADAS 2 assessment (Fig. 2) revealed a mixed risk of bias assessment regarding patient selection: A number of studies used only histopathology as the only reference standard, patient recruitment was non-consecutive or insufficient details regarding patient recruitment were given. In addition, benign lesions regularly contained only Bosniak IIF or Bosniak II and IIF cysts but no Bosniak I lesions [7–32]. No further risk of bias concerns was raised and all included studies were deemed applicable to answer the research question (Fig. 2). The study designs were described as prospective in one study [19] and retrospective in 33 studies [7–17, 20–41]. In one study [18], the retrospective or prospective character of the study could not be determined. Patient recruitment was consecutive in seven studies [13, 18, 19, 34, 39, 41]. Seven reports described non-consecutive [7, 8, 14, 24, 26, 36, 40] case-control patient recruitment. In another 21 studies, the consecutive or non-consecutive nature of patient recruitment was not clearly stated [9, 10, 12, 15–17, 20, 21, 23, 25, 27–33, 35–38]. Histopathology as a reference standard was used in 13 studies [7, 12, 15–17, 22, 28, 29, 31, 32, 35, 37, 41], follow-up and histopathology in another 21 studies [8–11, 13, 14, 18–21, 23–27, 30, 33, 34, 36, 38, 39], and, in one study only, follow-up was used as the reference standard [40]. Twenty-five of 35 (71.4 %) eligible studies provided technical information on computed tomography [7, 9–11, 13, 14, 16–19, 21, 23–27, 29, 31–33, 35, 36, 38, 39, 41] (Table 1). However, this information was incomplete in the majority of the investigated studies and almost all studies investigated their patients on several devices with varying protocols (Table 2). The number of observers reading CT images (range 1–3 readers) was provided in 21 studies [7–11, 13–15, 17, 19, 22–25, 27, 30, 32, 34, 36–38]. Observer experience (range 2–52 years’ experience) in CT was given in ten studies only [9, 10, 13, 14, 17, 19, 22, 23, 25, 32] (Table 1). Inter-observer variability based on kappa analysis (kappa range 0.571–1) was provided in five studies [13, 19, 23, 30, 34]. Eight studies were carried out before the introduction of the Bosniak IIF category [12, 28, 29, 33–35, 37, 38] and the remaining 27 studies after the introduction of Bosniak IIF [7–11, 13–20, 22–27, 30–32, 36, 39–41].

**Table 2:** Patient numbers, length of follow-up, CT equipment and reader experience in the included studies

First author and reference	Year	No. of patients	Length of follow-up* (mo)	CT vendor	CT detector rows	CT slice (mm)	Experience of readers (years)
Aronson [29]	1991	16	n.a	GE	1	n.a	n.a
Cloix [35]	1996	30	6–86	n.a	n.a	10	n.a
Wilson [38]	1996	20	n.a	GE	n.a	5–10	n.a
Siegel [34]	1997	46	n.a	n.a	n.a	n.a	n.a.
Bielsa [12]	1999	19	n.a	n.a	n.a	n.a	n.a
Curry [33]	2000	109	3–120	n.a	n.a	3–10	n.a.
Koga [37]	2000	35	n.a	n.a	n.a	n.a	n.a
Limb [28]	2002	57	6–70	n.a	n.a	n.a	n.a
Lang [31]	2002	22	67.2	GE/Philips/Siemens	n.a.	n.a	n.a
Israel, Bosniak [21]	2003	41	2–18	n.a	n.a	3–10	n.a.
Israel [11]	2003	81	13–209	n.a	n.a	3–10	n.a
Harisinghani [27]	2003	28	12–24	GE Medical Systems	n.a	5	‘Specialty-trained’
Israel [36]	2004	59	48	GE/Siemens		2.5–5	n.a
Spaliviero [41]	2005	50	14	Siemens	4; 16	≤5	n.a
Loock [20]	2006	37	n.a	n.a	n.a	n.a	n.a
Ascenti [19]	2007	40	12–24	Siemens	1; 16	3–5	15, 25, 10
Quaia [13]	2008	40	n.a	Philips/Toshiba	1, n.a.	3–5	2–8
Clevert [18]	2008	32	3–24	Siemens	64	≤3	n.a
Song [7]	2009	104	n.a	GE/Siemens	4; 16	2,5	n.a.
O’Malley [8]	2009	112	15	n.a	n.a.	n.a.	‘faculty’
Grotemeyer [40]	2009	25	n.a	n.a	n.a	n.a	n.a
Peng [39]	2010	22	n.a	GE	64	5	n.a.
Weibl [9]	2011	104	60	n.a		5	4–30
Pinheiro [15]	2011	36	n.a	n.a	n.a	n.a	n.a
You [16]	2011	53	n.a	GE, Siemens	4; 16	2.5	n.a
Smith [10]	2012	193	45,6 (IIF) 52,8(III)	Siemens	4–64	3–5	‘Fellowship-trained’
Han [17]	2012	97	n.a	GE	64	n.a.	10
Hwang [26]	2012	201	20	GE, Siemens	4; 16	2,5–5	n.a
Graumann [14]	2013	32	24–60	Siemens	4	2,5	5–20
Reese [22]	2014	133	n.a	n.a	n.a	n.a	n.a
Hindman [23]	2014	144	6–157	GE, Siemens	1; 4; 16; 64	3–8	7, 11, 52
Kim [25]	2014	164	24	GE, Siemens	16, 64	2.5–5	2–12
El-Mokadem [30]	2014	124	24	n.a	n.a	n.a	n.a.
Bata [32]	2014	19	n.a.	Philips	16	2	6
Weibl [24]	2015	85	43	n.a	n.a.	2.5–5	n.a.

**Table 3:** Reference standard and key diagnostic parameters extracted from the investigated studies

Author	Year	SOR	Diagnostic study: 0 = no, 1 = yes, 2 = yes, restricted to IIF benign	Lesions	TP	FN	FP	TN	Prevalence	95 % CI
Aronson [29]	1991	Histology	1	20	12	0	4	4	60.0	36.1–80.9
Cloix [35]	1996	Histology	1	32	11	2	12	7	43.3	23.7–59.4
Wilson [38]	1996	Histology/FU	1	22	10	4	0	8	93.3	40.7–82.8
Siegel [34]	1997	Histology/FU	1	70	31	1	9	29	66.7	33.8–58.1
Bielsa [12]	1999	Histology	1	20	10	1	2	7	55.0	31.5–76.9
Curry [33]	2000	Histology/FU	1	82	47	0	20	15	60.3	45.9–68.2
Koga [37]	2000	Histology	1	35	22	1	0	12	95.8	47.8–80.9
Limb [28]	2002	Histology	1	57	8	3	21	25	19.3	10.1–31.9
Lang [31]	2002	Histology	2	152	17	5	101	29	14.5	9.3–21.1
Israel and Bosniak [21]	2003	Histology/FU	0	42	0	2	0	40	4.8	0.6–16.2
Israel [11]	2003	Histology/FU	1	81	25	0	16	40	30.9	21.7–42.1
Harisinghani [27]	2003	Histology/FU	0	28	17	0	11	0	60.7	40.6–78.5
Israel [36]	2004	Histology/FU	1	69	20	0	8	41	37.0	18.7–41.2
Spaliviero [41]	2005	Histology	1	47	25	4	8	10	63.0	46.4–75.5
Loock [20]	2006	Histology/FU	1	37	10	2	11	14	32.4	18.0–49.8
Ascenti [19]	2007	Histology/FU	1	44	5	0	5	34	11.4	3.8–24.6
Quaia [13]	2008	Histology/FU	1	40	23	0	8	9	57.5	40.9–73.0
Clevert [18]	2008	Histology/FU	1	37	10	0	5	22	27.0	13.8–44.1
Song [7]	2009	Histology	1	104	53	3	22	26	53.8	﻿43.8–63.7
O’Malley [8]	2009	Histology/FU	2	107	27	5	6	69	29.9	﻿21.4–39.5
Grotemeyer [40]	2009	FU	0	25	0	0	0	25	0.0	0.0–13.7
Peng [39]	2010	Histology/FU	1	24	17	0	2	5	77.3	48.9–87.4
Weibl [9]	2011	Histology/FU	1	113	45	2	27	39	41.6	﻿32.4–51.2
Pinheiro [15]	2011	Histology	0	37	24	0	13	0	64.9	47.5–79.8
You [16]	2011	Histology	0	75	53	0	22	0	70.7	59.0–80.6
Smith [10]	2012	Histology/FU	2	213	58	4	86	65	29.1	23.1–35.7
Han [17]	2012	Histology	1	97	50	3	21	23	54.6	44.2–64.8
Hwang [26]	2012	Histology/FU	0	201	0	10	0	191	5.0	2.4–9.0
Graumann [14]	2013	Histology/FU	0	32	0	2	0	30	6.3	0.8–20.8
Reese [22]	2014	Histology	1	113	71	4	20	18	66.4	56.9–75.0
Hindman [23]	2014	Histology/FU	0	80	0	7	0	73	8.8	3.6–17.2
Kim [25]	2014	Histology/FU	1	164	52	6	16	90	35.4	28.1–43.2
El-Mokadem [30]	2014	Histology/FU	2	100	22	7	8	63	29.0	20.4–38.9
Bata [32]	2014	Histology	0	19	16	0	3	0	84.2	67.8–100
Weibl [24]	2015	Histology/FU	2	85	37	8	21	19	52.9	41.8–63.9

*SOR* standards of reference, *FU* follow-up

### Rate of malignancy in Bosniak categories

**Fig. 3:**
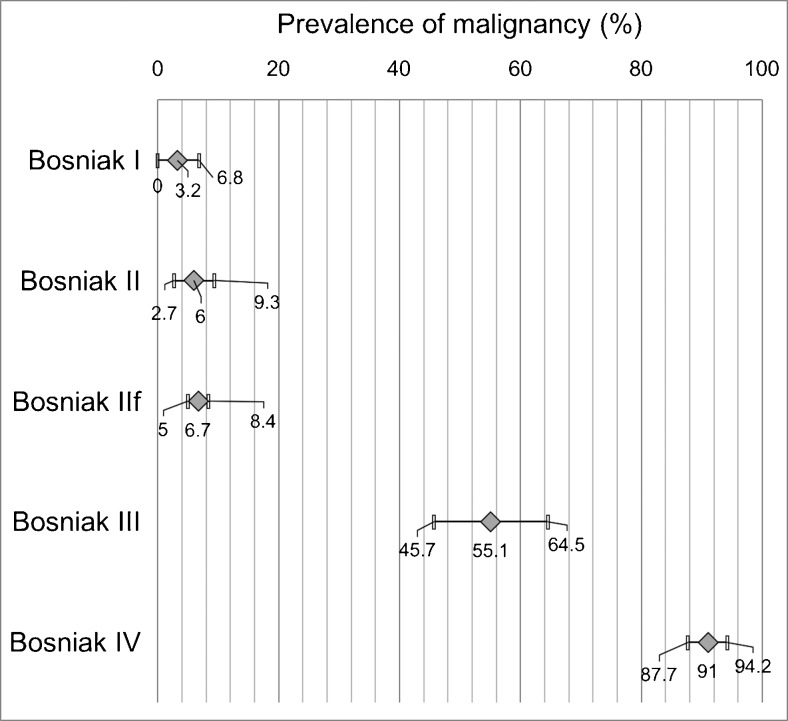
Forest plot of pooled malignancy rates (random effects model) in Bosniak categories

The rate of malignancy increased from Bosniak I to IV (Fig. 3 and Supplemental Material Fig. A1–A3). Pooled estimates were 3.2 % (95 % CI 0–6.8) in 89 Bosniak I, 6 % (95 % CI 2.7–9.3) in 261 Bosniak II, 6.7 % (95 % CI 5–8.4) in 818 Bosniak IIF, 55.1 % (95 % CI 45.7–64.5) in 887 Bosniak III and 91 % (95 % CI 87.7–94.2) in 449 Bosniak IV lesions. Malignancy rates did not differ between Bosniak I, II and IIF (P-values I vs. II: 0.309, II vs. IIF: 0.690, I vs. IIF: 0.199) and were higher in Bosniak III (P-values vs. IIF <0.0001) but lower than in Bosniak IV (P < 0.0001).

Two Bosniak I cysts were malignant: one an RCC upgraded by ultrasound [35] and one an incidental focal area (0.6 cm) of papillary RCC within a larger cyst [41]. Six studies provided details on benign Bosniak IV lesions: these were either smaller than 2 cm [17], haemorrhagic cysts [20, 35], cystic nephroma [15, 34, 39] or oncocytoma [15, 35], or simple cysts [35].

Meta-regression identified a higher rate of malignancy in Bosniak IIF lesions in studies that used histopathology as the only reference standard (16.6 %, 95 % CI 7.7–25.4), compared to studies that also accepted follow-up examinations as a reference standard (6.3 %, 95 % CI 4.6–8.0). Year of publication was associated with a trend towards higher malignancy rates (P = 0.05). No further influencing factors on the rate of malignancy were identified in any Bosniak category (P > 0.05, respectively). Between-studies heterogeneity was low in Bosniak I (I^2^ = 5 %) and IIF (I^2^ = 0 %), medium in Bosniak II (I^2^ = 32 %) and Bosniak IV (I^2^ = 36 %), and high in Bosniak III (I^2^ = 89 %) cysts.

### Diagnostic performance of the Bosniak classification

Twenty-six studies provided information about the diagnostic performance of the Bosniak classification by including benign and malignant lesions classified as benign (Bosniak < III) or malignant (Bosniak ≥ III) by imaging [7–13, 17–20, 22, 24, 25, 28–31, 33–39, 41].

**Fig. 4:**
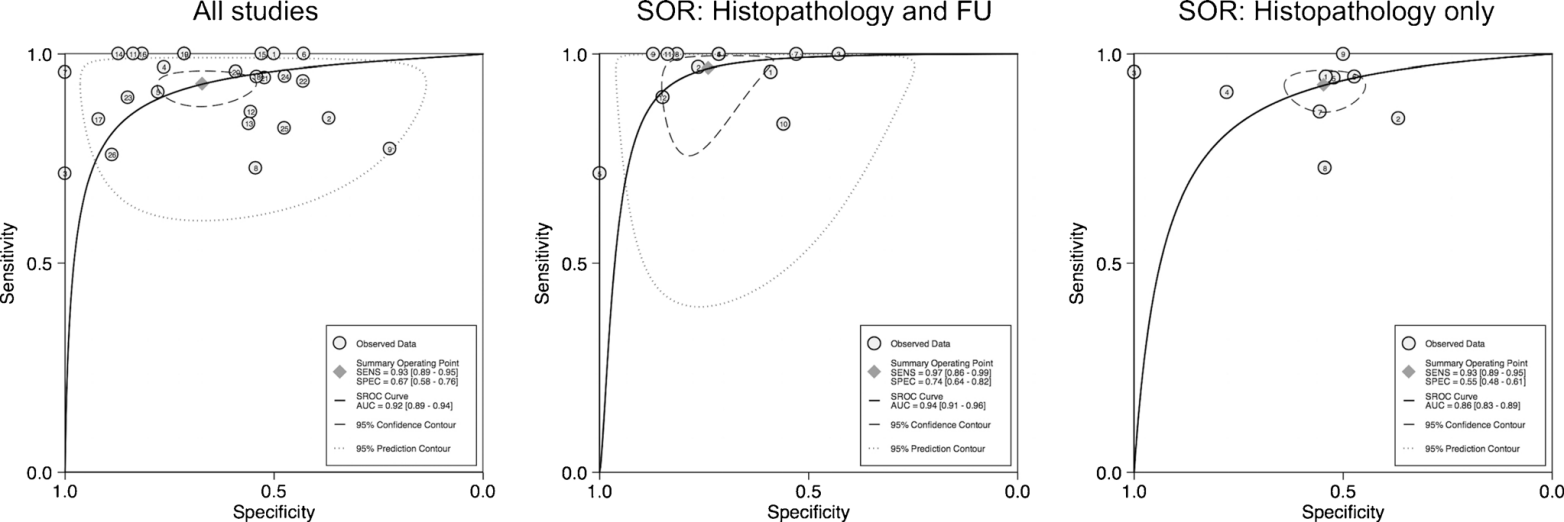
Summary receiver operating characteristic (ROC) curves based on bivariate (maximum likelihood) models for 26 diagnostic studies (left), a subgroup of 12 diagnostic studies with both histopathology and follow-up (FU) as standards of reference (SOR) (middle), and nine diagnostic studies using only histopathology as the SOR (right). Note a significantly (P < 0.001) higher area under the ROC curve (AUC) based on a higher (P < 0.001) specificity in studies with both histopathology and FU as the SOR, compared to histopathology only. The summary statistics in the middle most accurately reflect the clinical application of the Bosniak classification

The area under the summary ROC (sROC) curve (bivariate model) was calculated as 92 % (95 % CI 89–94; Fig. 4). Overall pooled sensitivity and specificity were 93 % (95 % CI 89–95) and 67 % (95 % CI 59–76). Between-study heterogeneity was high (I^2^ for sensitivity: 68.5 %, I^2^ for specificity: 90.9 %).

A subgroup analysis in diagnostic studies that included histopathology only as the standard of reference, and non-selected Bosniak categories [7, 12, 17, 22, 28, 29, 35, 37, 41], revealed a lower sROC AUC of 0.86 (95 % CI 0.83–0.89). A higher AUC of 0.94 (95 % CI 0.91–0.96) was found in 12 diagnostic studies that included histopathology and follow-up as the standards of reference [9, 11, 13, 18–20, 25, 33, 34, 36, 38, 39]. This group reflects the clinical setting most accurately, and a pooled (bivariate model) sensitivity of 97 % (95 % CI 86–99, I^2^ = 70.7 %) and a specificity of 74 % (95 % CI 64–82, I^2^ = 77.2 %) were calculated.

Meta-regression (random effects model) identified a lower sensitivity in studies that included only Bosniak IIF lesions as benign (meta-regression coefficient -0.76, 95 % CI -1.39 to -0.13, P = 0.018). Further, meta-regression demonstrated a significantly higher specificity in studies that used histopathology and follow-up as the reference standard as compared to studies with histopathology as reference standard only (meta-regression coefficient 0.92, 95 % CI 0.24–1.59, P = 0.008). Technical factors including slice thickness, detector rows or whether there was any technical information given at all did not show a significant influence on either sensitivity or specificity (P > 0.05, respectively). In addition, year of publication was not associated with these diagnostic performance indices (P > 0.05, respectively). No evidence of publication bias was found (Deek’s test P = 0.61; Supplemental Material Fig. B).

## Discussion

Our results demonstrate an increasing malignancy rate from Bosniak I to IV categories. Between-study heterogeneity ranged from low to high, with the highest value observed in Bosniak III lesions. Bosniak III cysts are defined as complex cysts and must be differentiated from minimally complex cysts (IIF) that can be managed with only follow-up. We did not identify any explanatory variable for the observed heterogeneity in Bosniak III lesions. However, two factors very likely contributing to this heterogeneity, namely, reader experience and spatial resolution, were insufficiently reported in the majority of included studies. Bosniak IIF cysts were more likely to be malignant if the study considered only histopathology as the standard of reference. As the rate of true-negative findings, and, subsequently, the malignancy rate, depends on whether clinically benign findings that are not subject to histopathological sampling are considered, a study design-related selection bias did appear to be present. The low to medium heterogeneity in Bosniak I, II and IV categories that was accompanied by a low (Bosniak I, II) or high (Bosniak IV) malignancy rate strongly suggests that the limitations of the Bosniak classification lie in a less-than-optimal grading of lesion complexity using the Bosniak IIF and III categories. This is underlined by the fact that the introduction of the Bosniak IIF category did not significantly affect the overall diagnostic performance of the Bosniak classification. In addition, the low but not very low malignancy rates of Bosniak II and IIF categories did not differ. These findings seem to suggest that Bosniak II lesions should be followed up in a similar way to IIF lesions. Again, selection bias might lead to an overestimation of malignancy rates in these lesions.

Overall, the Bosniak classification showed a sensitivity of 89.6 % and a specificity of 65.1 %. Meta-regression identified a lower sensitivity in studies that included only Bosniak IIF lesions as benign. This finding was attributed to the fact that there was a higher prevalence of malignancy in Bosniak IIF compared to the Bosniak II and I categories. Consequently, the rate of false-negative findings was higher per study design than in the case of a non-selected inclusion of all non-surgical Bosniak categories (I, II and IIF). In addition, study design-related specificity was lower in studies that used surgical verification only as the standard of reference. A higher rate of true-negative findings is to be expected when follow-up was used as the reference standard, as true-negative findings without subsequent surgery are not considered in a study considering histopathologically verified lesions only. In the latter case, specificity is expected to be lower. Consequently, we identified the best diagnostic performance for the Bosniak classification system in those studies most representative of the clinical setting: non-selected lesion inclusion and considering follow-up examination results in addition to histopathological work-up. Here, sensitivity was very high; conversely, the negative likelihood ratio was very low. This leads us to conclude that a negative Bosniak finding (Bosniak category < III) will sufficiently exclude malignancy. However, pooled specificity and positive likelihood ratios were rather mediocre. As false-positive findings regularly result in unnecessary treatment, or at least invasive diagnostic procedures, further research is needed to improve risk stratification and evidence-based clinical practice guidelines, especially for the management of Bosniak IIF and III findings. Accurate risk stratification would be a prerequisite for the adequate use of active surveillance strategies. However, our systematic review did not provide the data to resolve this issue.

The diagnosis of indeterminate cystic renal lesions may be improved by using additional imaging methods, such as contrast-enhanced ultrasound and magnetic resonance imaging (MRI). Contrast-enhanced ultrasound (CEUS) improves diagnosis by detecting fine enhancing septa and tumour vascularity in complex cysts [13, 42–44]. Similar diagnostic improvements can be obtained with MRI, which provides high soft-tissue contrast for the evaluation of septa and solid contrast-enhancing lesion parts. Israel et al. found a similar malignancy rate when comparing CT and MRI in 69 renal lesions. MRI had a tendency to upgrade the lesions: in 18 of 20 malignant lesions, CT and MRI agreed completely with regard to the Bosniak categorization, while MRI upgraded two CT Bosniak III lesions to Bosniak IV [36]. Chen et al. also compared CEUS and MRI of complex cystic renal masses and found a higher sensitivity and accuracy of CEUS (97.2 % and 84.5 %, respectively), but a lower specificity (71.4 %) versus 80.6 %, 78.9 %, and 77.1 %, respectively, for MRI [45]. These additional diagnostic tools have shown promising results with regard to lesion characterization. While CEUS is a relatively simple examination, MRI is considered rather time-consuming and expensive. In addition, minimally invasive percutaneous biopsies have a potential role in the management of renal cysts by separating surgical from non-surgical lesions and the value of this technique is currently under investigation. A detailed discussion on this topic is beyond the scope of this study, but there is a recent and comprehensive systematic review of new modalities for the diagnosis of complex renal cysts published by Ellimootill and co-workers [46].

Some limitations of our systematic review and meta-analysis warrant discussion. A majority of the studies included in our work provided insufficient data about technical and reading conditions. As most studies recruited their cases over a longer period of time, scanners and protocols were not kept constant. Extractable technical parameters and year of publication (assuming that year of publication and equipment are associated) did not show a significant influence on the diagnostic performance of the Bosniak classification according to our additional meta-regression analysis. Therefore, our analysis did not show a diagnostic impact of improved CT technology using the Bosniak classification for diagnosis of cystic renal lesions. However, as demonstrated in the results, technical influences on reader performance remain a research gap in this field. Further, observer experience and inter-observer variation were largely unexplored. As a consequence, there is an additional research gap regarding the rate of lesions with inconclusive or equivocal findings and the subsequent inability to determine a definitive Bosniak classification. In addition, the majority of studies were retrospective. Although we were able to identify several study design-related influences on malignancy rates and diagnostic parameters, a large amount of between-study heterogeneity remains unexplained. Again, these limitations should be seen as research gaps, highlighting where further research is necessary.

In conclusion, our meta-analysis provides quantitative summaries of malignancy rates in Bosniak categories. Strong heterogeneity in Bosniak IIF and III subgroups indicates the need for further research for improved clinical management of complex renal cysts. Considering studies most appropriately reflecting clinical practice, the Bosniak classification can accurately rule out malignancy, but its specificity remains moderate. The Bosniak classification is an accurate tool with which to stratify the risk of malignancy in renal cystic lesions and is seemingly robust along various protocols and CT scanner generations. Research gaps with regard to the clinical application of the Bosniak classification include a lack of data about reader experience and inter-reader variability, and the diagnostic influence of technical CT parameters.

## Electronic supplementary material

Below is the link to the electronic supplementary material.

ESM 1 (PDF 1901 kb)
