# Supplementary material for: Malignancy rates and diagnostic performance of the Bosniak classification for the diagnosis of cystic renal lesions in computed tomography – a systematic review and meta-analysis
Source: Eur Radiol. 2016 Oct 19;27(6):2239–47. doi: 10.1007/s00330-016-4631-9 (PMC5408031; doi:10.1007/s00330-016-4631-9)
Supplement: Supplementary file 1 — (PDF 1901 kb) [file 330_2016_4631_MOESM1_ESM.pdf]

Supplemental material

A) Forest plots of malignancy rates

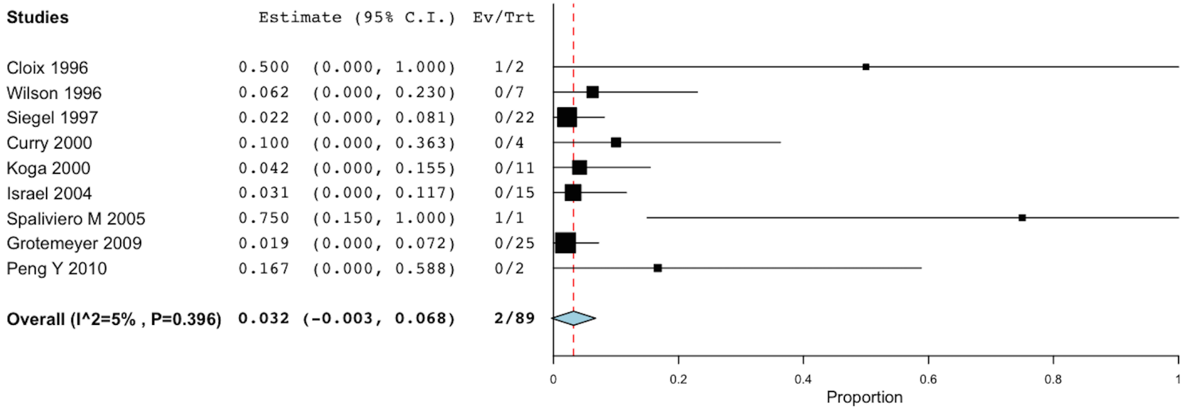

Figure A1: Pooled malignancy rates in Bosniak I cysts with corresponding 95%-confidence intervals.

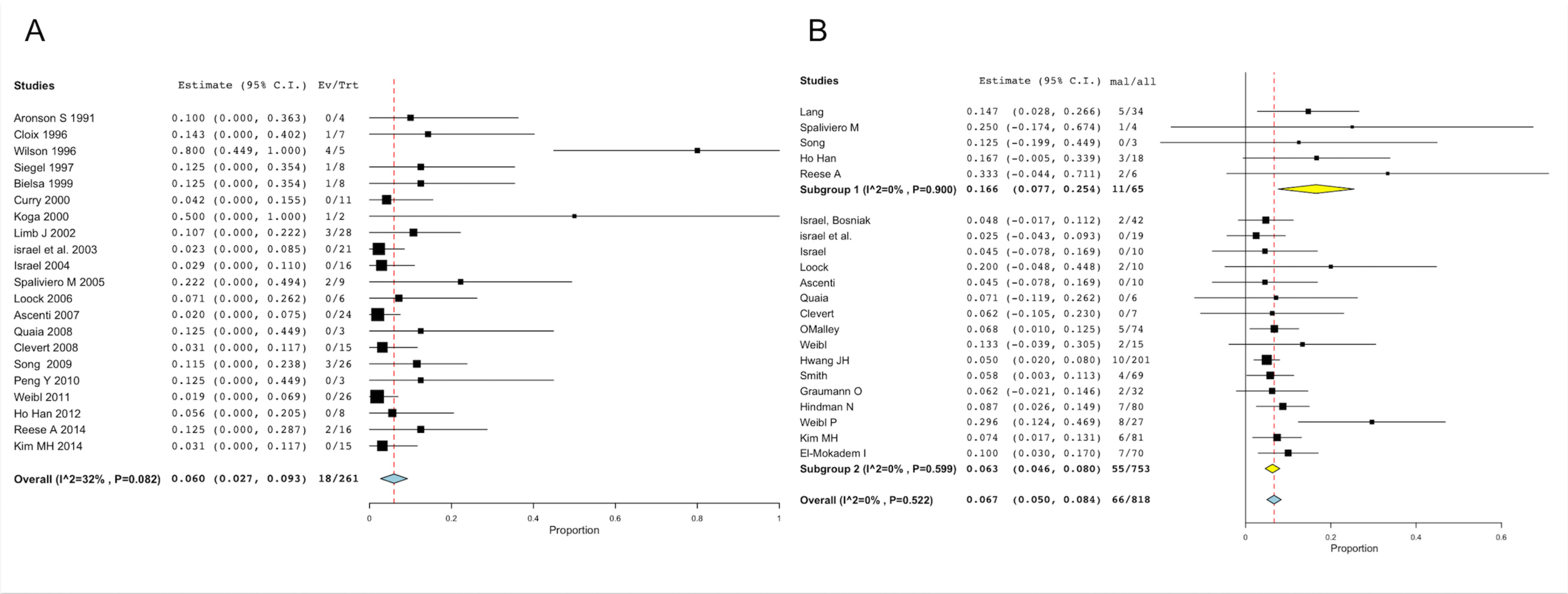

Figure A2: Pooled malignancy rates in Bosniak II (left) and IIF (right) cysts with corresponding 95%-confidence intervals.

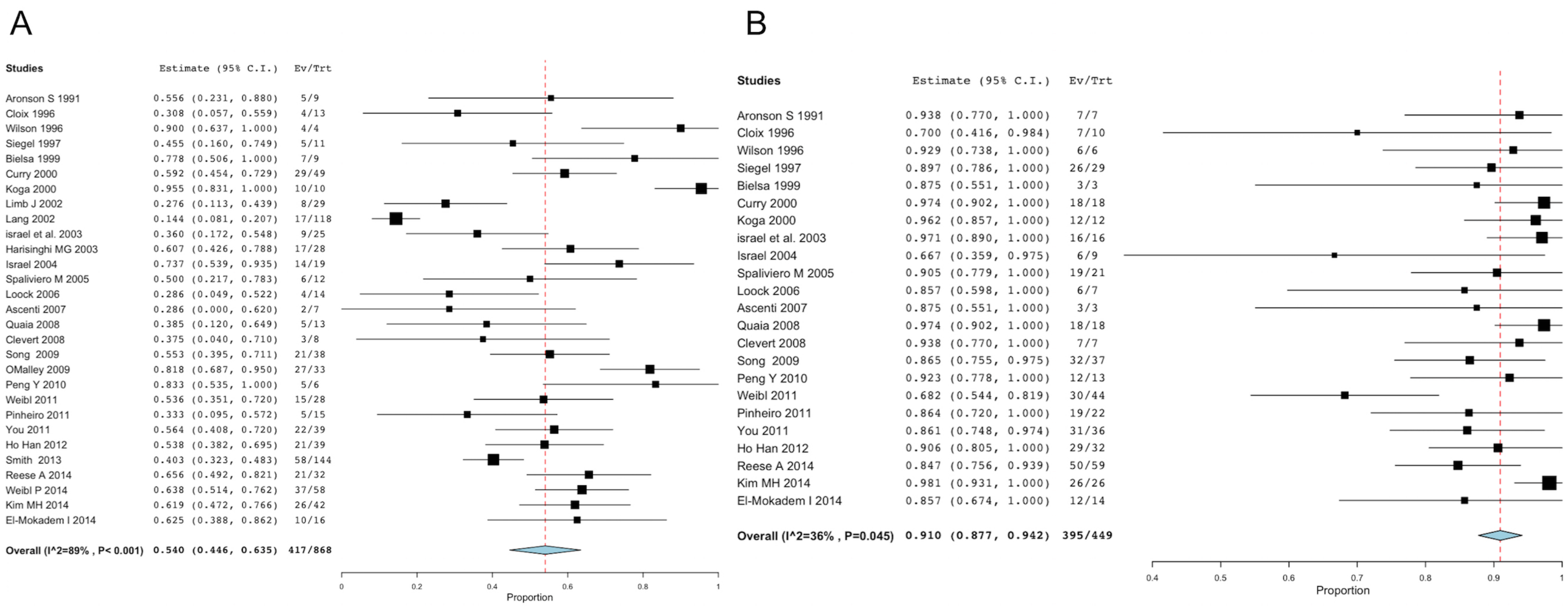

Figure A3: Pooled malignancy rates in Bosniak III (left) and IV (right) cysts with corresponding 95%-confidence intervals.

B) Funnel plots for the assessment of publication bias

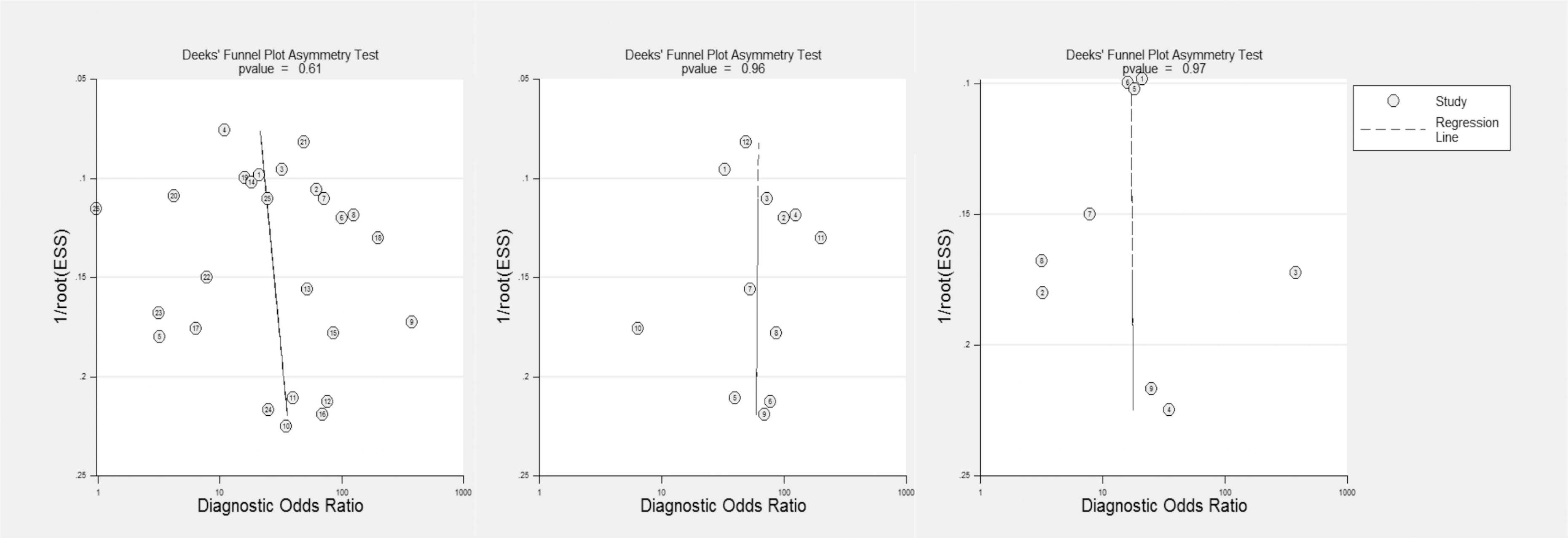

Figure B: Funnel plots for diagnostic odds ratios based on bivariate (maximum likelihood) model analysis of 26 diagnostic studies (left), 12 diagnostic studies with both histopathology and follow-up (FU) as standards of reference (middle) and nine diagnostic studies using only histopathology as the standards of reference. No evidence of publication bias was found.
